# Factor structure of Participation Behavioural Questionnaire (PBQ) in patients with hand injuries

**DOI:** 10.1371/journal.pone.0267872

**Published:** 2023-01-20

**Authors:** Maryam Farzad, Joy MacDermid, Mehdi Rassafiani

**Affiliations:** 1 School of Physical Therapy, Department of Health and Rehabilitation Sciences, University of Western Ontario, Ontario, Canada; 2 Roth McFarlane Hand and Upper Limb Centre, St. Joseph’s Hospital, London, Ontario, Canada; 3 Department of Occupational therapy, University of Social Welfare and Rehabilitation Sciences, Tehran, Iran; 4 Physical Therapy and Surgery, Western University, London, ON, Canada; 5 Clinical Research Lab, Hand and Upper Limb Centre, St. Joseph’s Health Centre, London, Ontario, Canada; 6 Rehabilitation Science McMaster University, Hamilton, ON, Canada; 7 Occupational Therapy Department, Faculty of Allied Health Sciences, Kuwait University, Kuwait City, Kuwait; 8 Paediatric Neurorehabilitation Research Centre, The University of Social Welfare and Rehabilitation Sciences, Tehran, Iran; ISCTE-Instituto Universitário de Lisboa: ISCTE-Instituto Universitario de Lisboa, PORTUGAL

## Abstract

**Background:**

Participation is considered a critical outcome of successful rehabilitation and should be evaluated.

**Objective:**

We aimed to evaluate the structural validity of the Participation Behaviour Questionnaire (PBQ) in people with hand injuries.

**Methodology:**

The PBQ contains 30 items that measure participation as conceptualized in the ICF. PBQ was developed with Rasch analysis to measure participation in hand injured. A sample of 404 patients with hand injuries and a mean age of 37 (16.0) participated and was randomly split for exploratory and confirmatory factor analysis (EFA/CFA).

**Results:**

Both EFA and CFA confirmed a four factor-solution. These factors were named: Social Participation and Interpersonal Relationships, Autonomy and Role, Subjective Satisfaction with Participation, Recreational, Sport, and Leisure Time. The value of Cronbach’s alpha was 0.96 for the total scale and >0.85 for each subscale.

**Conclusions:**

The structural validity of the PBQ was confirmed using both EFA and CFA. The PBQ measures four dimensions of participation.

## Introduction

Hand injuries are the second most common work-related musculoskeletal injury that leads to severe impairment [1]. Severe impairments cause disability and drastically affect the participation levels of a patient. Participation has been defined by the International Classification of Functioning, Disability and Health (ICF) [2] as an individual’s involvement in life situations. Based on the ICF, activity represents disability at the individual level and participation at the societal level [3]. Participation is using an activity to interact with others or with the environment. For instance, if we consider eating as an activity, eating with friends in a restaurant is participation. Any health condition can hinder involvement in life situations and thus restrict participation. Participation restriction is distinct from the outcome of activity limitation and impairment level and should be measured and targeted separately by the clinicians [4].

Participation contains different domains such as learning and applying knowledge; mental functions in general tasks and demands; communication; mobility; self-care; domestic life; interpersonal interactions and relationships; employment and economical life; and community, social, and civic life. The restriction of the participation domains is not the same in different populations with different disabilities. Different musculoskeletal conditions can cause different activity limitations, which will impact participation. For example, a patient with median nerve injuries will experience difficulties in picking up a coin or using a key (activity limitation) and restrict the participation level. Alternatively, one might expect patients with lower extremity impairments to have more mobility problems than those with upper extremity conditions. In contrast, patients with upper extremity issues might have more challenges manipulating, carrying, or interacting with technology. Different activity limitations profiles will also impact participation restrictions.

A consensus about measuring participation restriction as a different construct than activity limitation [5] has inspired the development of some generic questionnaires to measure participation. These generic questionnaires mainly measured interpersonal relationships and social participation [6–8]. Some participation questionnaires were also developed for specific conditions, such as brain injuries [9], distress [10], and hand injuries [11], which used the ICF framework and integrated patients’ reported restrictions in developing the questionnaires.

The Participation Behaviour Questionnaire (PBQ) [12] was developed to evaluate participation for patients with hand injuries using the ICF conceptual framework and considering patients’ reports of limitations in life situations and IRT methodology. The PBQ showed good measurement criteria regarding reliability, validity and unidimensionality [11, 13, 14]. The IRT/Rasch analysis confirmed the construct validity of the PBQ and reported it as a unidimensional measure. However, in measuring the construct validity, factor analysis is another method performed on all items of a PROM to evaluate the number of subscales of the PROM and the clustering of items within subscales (i.e., structural validity) [15]. In this study, we aim to explore the subscales underlying the board construct of participation with classic test theory via factor analysis [16]. Therefore, the purposes of this study were to evaluate the structural validity of the PBQ utilizing CTT and to confirm dimensionality as evaluated through IRT approaches.

## Materials and methods

### Measure

The Participation Behaviour Questionnaire (PBQ) [12] was originally developed in the Persian language to evaluate participation in patients with hand and upper extremity injuries. PBQ was developed using the ICF conceptual framework, considering patient’s reports of limitations in life situations and IRT methodology to evaluate participation comprehensively in nine domains, including domestic life; interpersonal interactions and relationships; major life areas; community, social and civic life, role, supporting others, domestic life, and subjective participation [11, 13, 14]. Rasch modelling was used to evaluate the psychometric properties, unidimensionality, validity and reliability by using the item and person separation indices, item fit, and potential for item reduction of the PBQ [17, 18]. PBQ showed a good fit to the Rasch model, suggesting that PBQ met the criteria for unidimensionality and confirmed good construct validity. Item reliability: 0.91, and Cronbach alpha: 0.96 indicated good reliability of the PBQ [12]. The PBQ contains a set of thirty self-reported items that ask the patients about the effect of the injury on their participation in important aspects of their life during the past weeks. Each item is rated on a numeric rating scale from totally disagree (score = 0, lowest) to strongly agree (score = 3, highest) [12]. The total score of PBQ is calculated by the summation of all items scores with the range from 0 to 90, with a higher score indicating more limitations in participation. Any questionnaires with more than 20% missing items cannot be used to evaluate participation.

### Participants and procedure

After approval by our local ethical review board, the PBQ was tested on a consecutive sample of 450 patients with different hand and upper extremity injuries. We included patients 18 years or older with any hand and upper extremity injuries in the past two months. All eligible patients were enrolled in an academic outpatient orthopedic clinic between March 2017 and May 2019. Patients with comorbidities such as chronic neurological disease (e.g., multiple sclerosis, stroke) were excluded. This study is a secondary analysis of an exciting database with an ethics approval number of IR.USWR.REC.1398.98 from the ethics committee of the University of Social Welfare and Rehabilitation Sciences, Tehran, Iran. After obtaining informed consent, patients completed the questionnaire.

### Statistical analysis

A sample size of 7 times the number of items and ≥100 samples is recommended for conducting FA from the COSMIN group is recommended for factor analysis; therefore, our sample size of 404 is sufficient to conduct the analysis [16]. Any questionnaires with more than 20% missing items were excluded from the analysis. Forty questionnaires with more than 20% missing items were excluded, and six patients declined participation, leaving 404 patients for final analysis. The sample of 404 participants was randomly split in half. The first half of the sample was used for the exploratory factor analysis, while the second half was used for the confirmatory factor analysis.

### Explanatory factor analysis

An exploratory factor analysis was performed to explore the factor structure and extract an underlying component measured by the PBQ. Sampling adequacy was measured with the Kaiser-Meyer-Olkin’s (KMO>0.8), and the correlation matrix was tested by Bartlett’s method (p<0.05) [19]. Factors were extracted using the Scree plot and Kaiser’s criterion (eigenvalues >1). Only significant loadings greater than or equal to 0.40 were used to interpret the factors [20]. Where Scree plot and Eigenvalues differed in factor solutions, multiple solutions were tested (i.e., dependent on Scree and Eigenvalue estimations), with the best factor solution chosen from the factor structure with the highest number of loadings, the highest number of stable factors, the lowest number of cross-loadings, and the lowest number of commonalities (the amount of variance accounted from that item to the factor) below 0.4. Following the EFA, Cronbach alpha values were examined from the derived factors to indicate the internal consistency of the items, with item exclusion for factors below 0.7 for factors with more than ten items and 0.5 for factors with fewer than ten items.

### Parallel analysis

We also used the Horn method parallel analysis to determine the number of factors by reducing the effects of sampling error on the eigenvalues method [21]. A data set was simulated with syntax written in SPSS, with a sample size of 205 and an item number of 30. The process was arranged with the iteration number of 1000, and the two data sets underwent parallel analysis. To decide on the number of factors, we chose the point that the eigenvalue of the simulative data was higher than the actual data [22]. The analysis was performed using SPSS software, version 23(Chicago, IL, USA). The significance level was considered as 0.05. The construct of each factor was interpreted and labelled based on the theoretical, subjective and inductive processes.

### Expert review

Three judges, individuals with expertise in occupational therapy, a fellow of the American Congress of Rehabilitation Medicine, and a Master of Public Health expert with expertise in the developed participation measures, were asked to review the resulting factor structure for content validity. The experts provided feedback regarding the conceptual definitions of the identified factors and items assessing each factor. Feedback from the reviewers informed the naming of the factors. Members of the expert panel were mailed a package containing a cover letter explaining the procedure and expectations, a detailed description of the researcher’s interpretation of the factors, and a complete list of the items retained from the exploratory factor analysis.

### Confirmatory factor analysis

Confirmatory factor analysis (CFA) was conducted to verify the factor structure developed based on EFA results with the second half of the sample [8]. Practical goodness-of-fit indexes were used to assess the model; comparative Fit Index (CFI ≥ 0.95), Goodness of Fit Index (GFI > 0.9), Tucker-Lewis index (TLI≥.95), and the root means square error of approximation (RMSEA: <0.05, good; <0.08, acceptable; >10, poor). The goodness of fit index (GFI) and Comparative fit index (CFI) was used to test the model fit, with larger values indicating better fit [23]. Data were analyzed with LISREL statistical software.

## Results

### Participants

A total of 404 participants completed the PBQ. The mean age of participants was 37 (16.0) years old, 32% were female, and 57% had an injury on their right hand. Demographics can be found in Table 1.

**Table 1:** Patients’ demographic characteristics (n = 404).

Demographic		Value
**Age: Mean (SD)**		37(16)
**Gender (n %)**	Female	125(31%)
	Male	279 (52%)
**Dominant hand (n %)**	Right	339(84%)
	Left	65(28%)
**Injured hand (n %)**	Right	289(74%)
	Left	115(36%)
**Diagnosis (n %)**	Compression neuropathies	48(12%)
	Soft tissue injuries	53(13%)
	Elbow and shoulder injuries	78(19%)
	Hand and wrist fracture	194(48%)
	Brachial plexus injuries	25(6%)
	Burns	6 (2%)

Continuous variables are presented as mean and standard deviation (range). Discrete variables are presented as percentages (number)

### Factor structure

**Fig 1:**
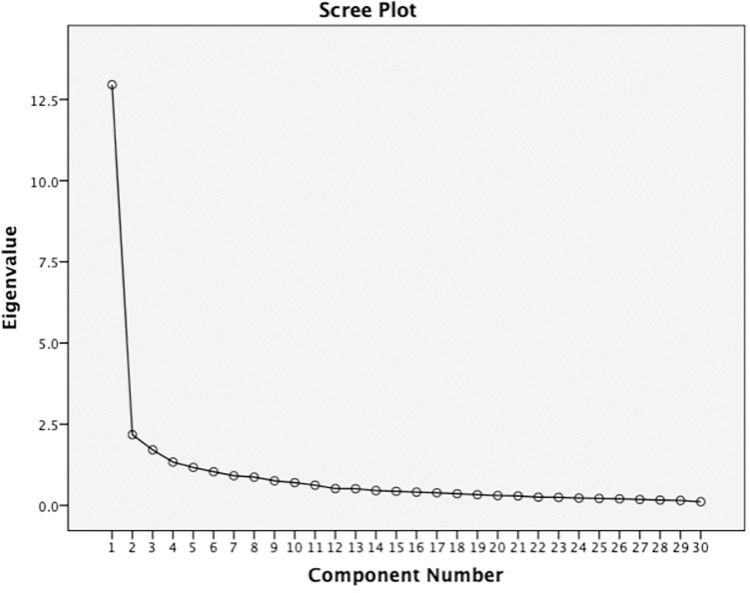
In this scree plot, the point at which the curve first begins to level off indicates that there is one dominant latent variable which is obviously dominant over the remaining latent variables.

The Kaiser–Meyer–Olkin test of sampling appropriateness was satisfactory (0.95), and Bartlett’s test of sphericity was statistically acceptable with [X2 = 264.092, p < .001]. The scree plot in Fig 1 shows that more than one component can be merged from the EFA. The eigenvalues explained variance and factor loadings for orthogonally and obliquely rotated two, three and four factors to explore the best factor structure. Table 2 displays the factor structure of the 30-item PBQ with all factor solutions after rotation.

**Table 2:** EFA of the PBQ; comparing three different factor solutions.

Items	Two factor solutions		Items	Three factor solutions			Items	Four Factor solution			
	Factor1	Factor 2		Factor1	Factor2	Factor3		Factor1	Factor2	Factor3	Factor4
**13**		0.79	**15**	0.75			**1**	0.71			0.47
**10**		0.76	**14**	0.72			**2**	0.70			
**30**		0.75	**29**	0.71			**3**	0.69			
**11**		0.72	**28**	0.71			**4**	0.68			
**16**		0.72	**5**	0.69			**5**	0.67			0.50
**12**		0.67	**3**	0.67			**6**	0.63			
**29**		0.67	**18**	0.65			**7**	0.61		0.45	
**27**		0.64	**17**	0.65			**8**	0.60		0.44	
**18**		0.63	**9**	0.61		0.57	**9**	0.48	0.47		
**8**	0.57	0.61	**8**	0.54			**10**	0.47			
**24**		0.61	**22**	0.46			**11**		0.82		
**25**		0.58	**2**		0.80		**12**		0.72		
**23**		0.57	**10**		0.73		**13**		0.64		
**14**		0.56	**13**		0.66		**14**		0.63	0.48	
**15**		0.53	**30**	0.49	0.64		**15**		0.62		
**17**	0.50	0.53	**16**		0.63		**16**	0.50	0.61		
**19**		0.52	**6**		0.62		**17**		0.60		
**2**	0.74		**11**		0.62		**18**	0.49	0.58		
**4**	0.73		**1**		0.61	0.55	**19**		0.54		0.44
**26**	0.73		**25**	0.51	0.59		**20**	0.41	0.53		
**7**	0.73		**20**		0.58	0.51	**21**			0.72	
**21**	0.72		**12**	0.51	0.52	0.40	**22**			0.69	
**1**	0.72		**27**	0.46	0.51		**23**	0.48		0.67	
**3**	0.71		**4**		0.47		**24**			0.58	
**5**	0.71		**13**			0.74	**25**			0.54	
**6**	0.69		**7**			0.65	**26**			0.50	
**9**	0.60		**23**	0.40		0.63	**27**			0.43	0.71
**22**	0.57		**21**		0.45	0.61	**28**	0.51			0.68
**20**	0.53		**24**			0.60	**29**				0.67
**28**	0.53		**26**	0.45		0.58	**30**				0.56
**Alpha**	**0.92**	**0.94**		**0.92**	**0.93**	**0.87**		**0.91**	**0.90**	**0.87**	**0.85**
**Variance%**	**48.39**	**6.86**		**48.39**	**6.86**	**4.90**		**48.39**	**6.86**	**4.90**	**4.22**
**Eigenvalue**	**14.52**	**2.05**		**14.51**	**2.05**	**1.47**		**14.51**	**2.05**	**1.47**	**1.27**

All the models were significant, but the variances accounted for data were higher in the four factors solution (64.40%).

We intended to use parallel analysis to provide further evidence or a basis for deciding the number of factors more easily. The results showed that the eigenvalue of the first factor in the actual data was 14.52, while it was 1.91 in the simulative data set. The second factor’s eigenvalue in the actual data was 2.06, whereas it was 1.78 in the simulative data. The eigenvalue of the third factor in the actual data was 1.47, whereas it was 1.66 in the simulative data. The fourth factor’s eigenvalue in the actual data was 1.27, while it is 1.58 in the simulative data. When we shifted from the fourth factor to the fifth, the case was different, and thus the number of the scale factors is determinedly restricted to 4 because the eigenvalue of the simulative data (1.52) of the fifth factor was higher than the actual data (0.94).

**Fig 2:**
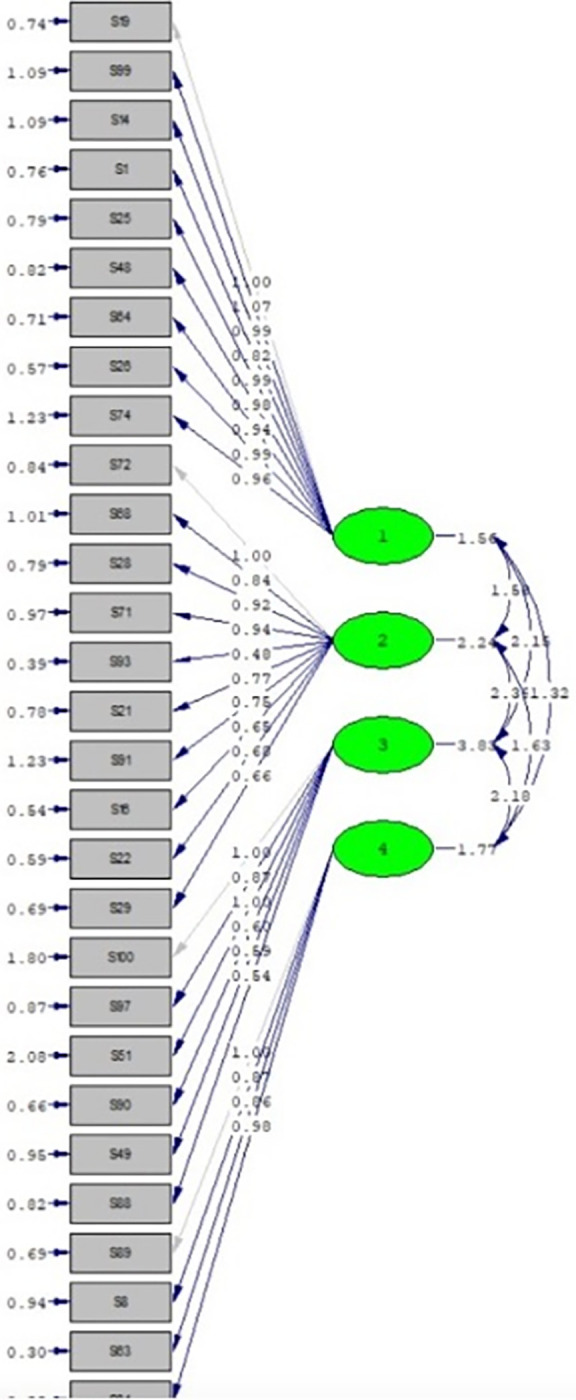
Four factor structure of the PBQ based on the results of CFA. Four factors were retrieved from the FA and can be seen as 1 to 4, which were named as follow: Social Participation and Interpersonal Relationships, Autonomy and Role, Subjective Satisfaction with Participation, Recreational, Sport, and Leisure Time. Each subscale was formed with a group of items from the original questionnaires, which can be seen in gray rectangles. All the subscales are intercorrelated, as can be seen with arrows among subscales. The correlation between each item and the subscale can be followed with the arrows.

The derived EFA factor model was entered into a CFA using the second half of the sample. The CFA supported four subscales that fit the four described subscales of the PBQ. A non-significant item trait interaction (chi-square, 3759.49, P<0.001, df = 406), relative chi-square) (chi-square index divided by the degree of freedom more than 2 (9.25) and RMSEA less than 0.08 (0.070) empirically confirmed verification of the four-factor structure solution. GFI (0.96), CFI (0.99), and TLI (0.96) indices more than 0.9 in the four-factor structure model indicated a good fit to the data (Fig 2).

Based on the four-factor solution, names were assigned to the factors based on the recommendations from the experts, and internal consistency was tested for each factor. We emailed the final four-factor structure questionnaire to the invited experts and asked them to name each factor. The research team finalized the factors’ names and concluded on the suggestions. Factor one included ten items with loading values between 0.56 and 0.74 and reported Cronbach’s alpha of 0.91. The first factor, which explains 48.39% of the common variance of PBQ, with ten items, contained items that measured interpersonal relationships and social participation; it was entitled based on the recommendations from experts as "social participation." The second factor included ten items with loading values between 0.43 and 0.44 and a Cronbach’s alpha of 0.90. Factor two evaluates the individual’s assessment of their functional ability to participate, and so it was entitled "Autonomy and role." The third factor included six items with loading values between 0.40 and 0.76 and Cronbach’s alpha of 0.85. Factor 3 assessed the degree to which the individuals feel their participation is accepted by others, are satisfied with their participation and entitled to "Subjective satisfaction with participation." Factor 4 included four items with loading values between 0.47 and 0.63 and Cronbach’s alpha of 0.85. Factor 4 assessed the individual’s assessment of their ability to participate in recreational activities and was entitled, "Recreational, sport, and leisure time." Cronbach’s alpha was 0.96 for the total scale of 30 items (Table 2).

### Final PBQ

The final PBQ contains four subscales: Social Participation and Interpersonal Relationship, Autonomy and Role, Subjective Satisfaction with Participation, and Recreational, Sport, And Leisure Time. Subscales 1 and 2 have ten items. Subscales3 includes seven items, and the fourth has four items with scores ranging from 0 to 3. The mean score of total PBQ in our sample was 85.15 (SD: 23.27). All subscales and scales achieved acceptable internal consistency (alpha: 0.85–0.91) (Fig 2).

## Discussion

This study provides additional information regarding the structural validity of the PBQ using factor analysis to extract the factor structure of the PBQ derived from Classic Test theory (CTT) approaches. CTT and IRT differ significantly in their modelling processes, making fundamentally different assumptions about the nature of the measured construct [24, 25].

Item response theory with Standard Rasch analysis is based on unidimensional models where a single latent trait is assumed to determine individuals’ performances on the test. The questionnaire could be analyzed for unidimensionality as a whole or separately for each subscale. The first approach cannot retrieve the subscale structure of the test, and the second approach ignores the potential intercorrelations between related but not identical latent traits [25, 26]. In the development of PBQ, the unidimensionality was tested on the whole questionnaire. The result confirmed that the PBQ measures the construct of participation, and no latent trait exists. The CFA demonstrated that a four-factor solution provided the best fit. We asked invited experts to label each factor considering the content of the items in each factor.

The research team then finalized the labelled: Social Participation and Interpersonal Relationships, Autonomy and Role, Subjective Satisfaction with Participation, and Recreational, Sport, and Leisure Time.

The first factor contained items that evaluate social participation. Based on definitions, the core central theme of participation is the social aspect of participation and interpersonal relationships. Social participation is an essential domain of participation which can discriminate participation from activity. Based on the ICF definition, participation is involvement in life situations [27]. However, in the ICF classification system, activity and participation are defined separately but included under the umbrella term of disability, with "d" codes used to specify the specific areas of disability, which leaves some conceptual ambiguity between activity and participation (28). Multiple authors have tried to further refine definitions of participation in ways that discriminate participation from activity. Operational definitions of participation have alluded to: fulfilling social roles and performance at the societal level [29], active engagement in intrinsically social activities [30], purposeful things that people do and defined by a social role (societal involvement) [28], societal involvement [29–31], being with others, interacting with others, doing an activity with others, helping others [32]. Some authors indicated the specific chapters of the ICF from d chapters; including assisting others (d660), particular interpersonal relationships (d730–d779), education (d810–d839), work and employment (d840–d859), economic life (d860–d879), community life (d910), recreational and leisure (d920), religion and spirituality (d930), and political life and citizenship (d950) can be considered as participation [29].

The second factor included items that evaluated ’Autonomy and Role’ and explained 6.86% of the variance of PBQ. Furthermore, role performance [33] is an important defining characteristic of participation considered in PBQ. Role performance implies that the individual is executing and experiencing their specific role, such as being a student, mother, etc. [34]. This also can differentiate participation from activity [28, 35].

The third factor contains items that measure the Subjective satisfaction with participation which is the internal aspect of participation, commonly defined as subjective or perceived participation. The ICF does not cover satisfaction with participation and engagement as the definition of ICF focuses on functioning [3, 30], thus may not capture some important perspectives about participation, including satisfaction and engagement. A qualitative study based on an interview with disabled patients identified important aspects of participation such as active and meaningful engagement/being a part of choice and control [3]. Involvement and engagement are subjective subdimensions of participation often neglected in measuring participation [34]. Based on the definition, engagement comprises the individual’s behavioural, cognitive, and affective investment during role performance [34]. However, the PBQ contains items that capture behavioural, cognitive, and affective dimensions of participation and measure satisfaction with participation.

The fourth factor evaluates participation in recreational, sports, and leisure time. Recreational and leisure (d920) is a chapter of ICF recommended as participation and represents the major participation domain of ICF [29].

The content of four extracted subscales indicated that the PBQ could evaluate participation comprehensively in patients with hand injuries. Many standard outcome measures are used in practice and specially designed to evaluate participation; however, their items do not fully capture the construct of participation in patients with hand injuries [36]. There are also some generic measures to evaluate participation [37–39]. The results from a systematic review of the content of existing generic participation measures indicated that no existing participation measure covers the complete set of participation domains identified in the ICF, and different PROMs address different domains of participation. They also reported that many PROMs measure a mixture of concepts (e.g., activity, symptoms) and participation [31, 40]. Some outcome measures evaluate disability in hand injured patients, which contains some items that measure participation; however, there is no specific outcome measure to evaluate participation [13, 41, 42].

The PBQ is a valid PRO that can be used to assess participation across the different domains of ICF, including domestic life, interpersonal interactions and relationships, major life areas, community, social and civic life, role, supporting others, domestic life, and subjective participation. As the PBQ contained items based on the patients’ perspective, it has items related to subjective participation and satisfaction [30, 43–45] and personal preferences in participation [29], which are not considered by ICF in participation coding. The factor analysis of the PBQ showed that it is better to define participation using four separate subscales (social participation and interpersonal relationships, autonomy and role, subjective satisfaction with participation, recreational, sport, and leisure time) rather than as a single summative score.

Additional psychometric analyses using newer techniques, such as those drawn from Rasch or item response theory, to evaluate the unidimensionality of each subscale, targeting and different item functioning, and item difficulty is warranted. Evaluating the longitudinal Validity of the PBQ and its subscales is needed to know the responsiveness of the PBQ in detecting changes over time.

There are some key limitations to interpretation that should be observed. First are the missing items and uncomplete data sets. While most studies would be satisfied with greater than 80% retention, it is worth recognizing that uncompleted items represent a small but important subset of the information. As the data were collected via questionnaire and each participant independently completed the questionnaire, it was impossible to determine the reasons for skipping items. We considered lack of attention, unclear wording, or participants’ discomfort in answering certain items as possible reasons for missing items. Our considerable early work on the first version of the PBQ, which included cognitive debriefing and confirming its content validity, would suggest that unclear wording is an unlikely problem. Another limitation of this study is that PBQ is a multi-dimensional instrument that is scored on an ordinal scale which is difficult to interpret. The content range is unclear, and caution must be considered when interpreting scores.

## Supporting information

S1 File (DOCX)
