# Supplementary material for: Factor structure of Participation Behavioural Questionnaire (PBQ) in patients with hand injuries
Source: PLoS One. 2023 Jan 20;18(1):e0267872. doi: 10.1371/journal.pone.0267872 (PMC9858033; doi:10.1371/journal.pone.0267872)
Supplement: S1 File — (DOCX) [file pone.0267872.s001.docx]

| Participation Questionnaire | | | | | |
| --- | --- | --- | --- | --- | --- |
| **Social Participation and interpersonal relationship** | | | | | |
|  | | Strongly disagree | Disagree | Agree | Strongly agree |
| 1 | My social activities are reduced (participation in group work, etc.). | 0 | 1 | 2 | 3 |
| 2 | I cannot participate in public places |  |  |  | 3 |
| 3 | I cannot communicate with my colleagues or co-workers |  |  |  | 3 |
| 4 | I can not get or keep a paid or voluntary job. |  |  |  | 3 |
| 5 | I cannot help others. |  |  |  | 3 |
| 6 | I can no longer visit with my relatives. |  |  |  | 3 |
| 7 | I cannot communicate with my friends like before. |  |  |  | 3 |
| 8 | I cannot entertain my relatives and friends in my home. |  |  |  | 3 |
| 9 | I mostly try to communicate indirectly (by phone, email, etc.) with others. |  |  |  | 3 |
| 10 | I cannot use public transportations. |  |  |  |  |
| **Autonomy, and role** | | | | | |
| 1 | I cannot fulfill my role at home. |  |  |  | 3 |
| 2 | I cannot look after my family |  |  |  | 3 |
| 3 | I feel I have lost my autonomy |  |  |  | 3 |
| 4 | I cannot take care of my self |  |  |  | 3 |
| 5 | I cannot handle my house works. |  |  |  | 3 |
| 6 | I can no longer look after my home. |  |  |  | 3 |
| 7 | I do not have mastery in doing my daily routines outside of home |  |  |  | 3 |
| 8 | I cannot do my self-care independently |  |  |  | 3 |
| 9 | I have difficulty in moving around |  |  |  | 3 |
| 10 | I cannot cope with my functional problem. |  |  |  | 3 |
| Subjective satisfaction with participation | | | | | |
| 1 | My family members are avoiding me. |  |  |  | 3 |
| 2 | The people’s desire to communicate with me has been decreased. |  |  |  | 3 |
| 3 | It is difficult for me to tolerate the reaction of others to the appearance of my hand. |  |  |  | 3 |
| 4 | I feel incompetent like I can no longer participate the way I used to |  |  |  | 3 |
| 5 | I feel dependent on others for doing many of my tasks. |  |  |  | 3 |
| 6 | I feel uncomfortable that my behaviour and movements seem inconsistent compared to others. |  |  |  | 3 |
| Recreational, sport, and leisure time | | | | | |
| 1 | I have fewer sport activities than before. |  |  |  | 3 |
| 2 | I cannot participate in entertainment and recreational activities. |  |  |  | 3 |
| 3 | My religious activities have been diminished. |  |  |  | 3 |
| 4 | My entertainment and amusement activities have been limited. |  |  |  | 3 |
